# An improved gray prediction model for China’s beef consumption forecasting

**DOI:** 10.1371/journal.pone.0221333

**Published:** 2019-09-06

**Authors:** Bo Zeng, Shuliang Li, Wei Meng, Dehai Zhang

**Affiliations:** 1 College of Business Planning, Chongqing Technology and Business University, Chongqing, PR China; 2 Collaborative Innovation Center for Chongqing‘s Modern Trade Logistics & Supply Chain, Chongqing Technology and Business University, Chongqing, PR China; Rutgers University, UNITED STATES

## Abstract

To balance the supply and demand in China's beef market, beef consumption must be scientifically and effectively forecasted. Beef consumption is affected by many factors and is characterized by gray uncertainty. Therefore, gray theory can be used to forecast the beef consumption, In this paper, the structural defects and unreasonable parameter design of the traditional gray model are analyzed. Then, a new gray model termed, EGM(1,1,*r*), is built, and the modeling conditions and error checking methods of EGM(1,1,*r*) are studied. Then, EGM(1,1,*r*) is used to simulate and forecast China’s beef consumption. The results show that both the simulation and prediction precisions of the new model are better than those of other gray models. Finally, the new model is used to forecast China’s beef consumption for the period from 2019–2025. The findings will serve as an important reference for the Chinese government in formulating policies to ensure the balance between the supply and demand for Chinese beef.

## Introduction

Beef has a rich protein content and low fat content. Moreover, the amino acid composition of beef is much closer to human needs than that of pork and can improve the body's resistance to disease. Hence, beef has gradually become one of the most popular meat products in China [1]. Since China is the most populous country in the world, a small increase in the proportion of beef consumption will lead to a substantial increase in total beef consumption in China. Therefore, scientifically and effectively predicting the level of Chinese beef consumption over the short and long terms and grasping the developmental trend and overall scale of Chinese beef consumption are of great value to the Chinese government. The results can help the Chinese government adjust the supply of domestic beef, formulate beef import and export policies, promote the effective supply of beef, and ensure the balance of supply and demand in the Chinese beef market and the healthy development of the beef industry.

The gray predictive method is one of the most important time series predictive methods, and it has been used in solving uncertainty problems with small data and poor information. The gray forecasting models have been applied in many fields such as agriculture, society, energy, industry, economics, and ocean research. And this predictive method has successfully solved a large number of issues in management, production, and uncertainty research. With the rapid progress of application for gray forecasting method, the traditional gray model (1,1) was improved into a lot of forecasting model types such as GM(1,N)(Gray Model with n variable and one first-order equation), GM(0,N) (Gray Model with n variables and zero-order equation), GM(2,1) (Gray Model with one variable and one two-order equation),DGM(1,1) (Discrete Gray Model with one variable and one first-order equation),the Verhulst model, SAIGM(Self-Adapting Intelligent Gray Model),etc. All of them are trying to solve the problem of the prediction and simulation accuracy[2–5]. According to the characteristics of small sample data of beef consumption in China, we built EGM(1,1,r)(the even form of the Gray Model with one variable and one first-order equation with the order r of the accumulation generation) model to solve the above problems.

## Data characteristics and method selection

The total beef consumption in a country or region is affected by many factors[6–10], such as the total population, the age structure, the income per capita, the relative price of beef, the quality and variety of beef, the consumption habits of the residents, the attitudes and preferences of the consumers, etc. Some of these factors can be quantified, such as the total population and the income per capita, while others are difficult to quantify, such as the consumption habits of residents. The mathematical statistical model is a common prediction model [11–14]. This model is mainly based on the relationship between the dependent variable (the amount of beef consumption) and the independent variables (population, income per capita, etc.), and the prediction of the dependent variable is realized on the assumption that the change trends of the independent variables are known first. This assumption creates great uncertainty in the accurate prediction of dependent variables because it is difficult to know the exact data for some independent variables[15–17]. Because the factors affecting the total beef consumption are complex and some indexes are difficult to quantify, it is difficult to forecast beef consumption using the traditional causality prediction model [18–22].

On the other hand, the statistical data about China’s beef consumption is limited. China proposed the reform target of creating a ‘socialist market economy’ in 1991 and has gradually achieved the transformation from a planned economy to a market economy. This transformation stage has been called ‘the transition period of China’s market economy’ by the United States. In 2001, China became a ‘market economy’ country, based on China joining the WTO (World Trade Organization). Before China formally joined in the WTO (i.e., before 2001), beef consumption in China had the typical consumption pattern in a planned economy guided by the central government. In other words, at that time the output and consumption of beef in China were controlled from the top down by government planning. The government decided important factors such as the beef production yields and the allocated uses each year. Hence, the data obtained before 1991 have little referential value for studying the beef consumption in the current market economy environment.

Therefore, the statistical data concerning beef consumption from 1991 to 2018 serves as the modeling sample in this study. Meanwhile, in order to verify the model’s predictive performance, the real data for 2016–2018 will be used as the benchmark data for evaluating the model’s performance. Thus, only 25 data points, i.e., those for 1991–2015, are available for use in building the model, with the resulting characteristic of a ‘small sample’. Hence, models with large samples, such as the ARIMA(autoregressive integrated moving average model), RBFNN(radial basis function neural networks), ELM(extreme learning machine) and SVM(support vector machine) [23–29] are not suitable. Because the stability of 25 data points cannot guarantee that it can continue along the existing state "inertia" in the future, the mean and variance of the data series will change obviously when the data points are less than 30. Moreover, the residuals of the 25 data are far from each other and not in the same line, so it is meaningless to test them. For example, no matter how to test the 25 samples, the p value is greater than 0.05.

The gray prediction model is a commonly used method to study uncertain system prediction problems with small samples [30,5]. The method considers the fact that the evolution of a system is affected by many complex factors (the factors are uncertain, hence the name ‘Gray Factor’), in which any tiny change in a factor may have a great influence on the variation of the dependent variable (the butterfly effect), and in which the change trend of the dependent variable is the result of the influence of many complex factors[4]. In other words, the dependent variable itself already contains the effect of many factors, and it is formally expressed as a certain number. (When the result is certain, it is referred to as a ‘White Result’.) Therefore, we can excavate the evolutionary trend of the dependent variable according to the variations of the dependent variables and then realize the prediction of the future developmental trend of the system[31].The total consumption of beef is affected by many factors. The existing statistical analysis for factors is the method of correlation and regressions usual, but the premise is that the size of sample is large enough and the sample has to fit typical distribution, its calculation process is relatively complicated. It is very difficult for modeling when the relationship between independent variable and dependent variable is nonlinear. Although Markov model needs few amount of data, its calculation accuracy is low and storage complexity is high. Grey prediction models which is based on GM(1, 1), and DGM(1, 1) model build exponential function to realize predicting mainly through the generation accumulating of raw data sequence, but its accuracy of simulation and prediction is not ideal. Because index form has the monotonicity, its change rule does not conform to the oscillation characteristics of the original sequence. Although a self-adapting intelligent gray prediction model (SAIGM) can automatically optimize parameters and select a reasonable model structure to adapt to the real data characteristics of the modeling sequence, the mean relative simulation percentage error(MRSPE) of SAIGM model still high sometimes.

In this paper, the gray system model is used to simulate the Chinese beef consumption, which has the characteristics of a ‘Gray Factor White Result’. However, although the gray prediction model has made great progress in its modeling mechanism and performance optimization since the 1980s[3, 32] and some new practical gray system models have been developed [33–34], there are still some problems with the structural and parameter optimization of the traditional gray prediction model [35–40]. Therefore, an improved gray model termed, EGM(1,1,*r*), is built, and the modeling conditions and error checking methods of EGM(1,1,*r*) are studied. Then, EGM(1,1,*r*) is used to simulate and forecast China’s beef consumption.

## Paper structure and tables of notation

Following the sequence of proposing problems, improving the methods, solving the problems and analyzing the conclusions, the paper is organized as follows. In Section2, we introduce the definition of the classic gray EGM(1,1) model (as shown in Table 1 the same as bellow)and then analyze its structural defects and the unreasonable design of the accumulating order. In Section 3, we propose an improved new gray prediction model, EGM(1,1, *r*) (as shown in Table 1 the same as bellow), introduce the parameter estimation and optimization method, and deduce the time response function of EGM(1,1, *r*). In Section 4, we introduce the modeling conditions and error testing methods of the new EGM(1,1, *r*) model. In Section 5, we mainly use the EGM(1,1, r) model to simulate and forecast the total Chinese beef consumption. The main contents include the modeling condition test, the parameter calculations and the optimization of the EGM(1,1,r) model; the simulation performance comparisons between EGM(1,1, r) and other gray models;, and the prediction analysis of the total Chinese beef consumption. In Section 6, we summarize our conclusions and introduce the research work to be carried out in future research.

**Table 1:** Abbreviations and corresponding definitions for different gray prediction models[4].

Index	Abbreviation	Definition
1	GM(1,1)	Gray Model with one variable and one first order equation
2	UGM(1,1)	Unbiased Gray Model with one variable and one first-order equation
3	DGM(1,1)	Discrete Gray Model with one variable and one first-order equation
4	EGM(1,1)	The even form of the Gray Model with one variable and one first-order equation
5	EGM(1,1,r)	The even form of the Gray Model with one variable and one first-order equation with the order r of the accumulation generation
6	SAIGM	Self-Adapting Intelligent Gray Model

To have a full understand of notations in this paper, we draw two tables of notation as follows.

## EGM(1,1)model and its imperfections

**Definition 1**[30]. Let *X*^(0)^ = (*x*^(0)^(1),*x*^(0)^(2),⋯,*x*^(0)^(*n*)) be a nonnegative original sequence, where *x*^(0)^(*k*)≥0 and, *k* = 1,2,⋯,*n*. Then, *X*^(1)^ is the 1-AGO (accumulation generation operator) sequence of *X*^(0)^ (as shown in Table 2,the same as bellow) the following:

$$X^{\left(1\right)}=\left(x^{\left(1\right)}\left(1\right),x^{\left(1\right)}\left(2\right),\cdots ,x^{\left(1\right)}\left(n\right)\right)$$

where

In addition *Z*^(1)^ is the mean generated sequence of consecutive neighbors of *X*^(1)^ as follows:

$$Z^{\left(1\right)}=\left(z^{\left(1\right)}\left(2\right),z^{\left(1\right)}\left(3\right),\cdots ,z^{\left(1\right)}\left(n\right)\right)$$

$$Z^{\left(1\right)}=\left(z^{\left(1\right)}\left(2\right),z^{\left(1\right)}\left(3\right),\cdots ,z^{\left(1\right)}\left(n\right)\right)$$

where

$$z^{\left(1\right)}\left(k\right)=0.5x^{\left(1\right)}\left(k\right)+0.5x^{\left(1\right)}\left(k-1\right),\;k=2,3,\cdots ,n$$

**Definition 2.** [30] Let *X*^(0)^,*X*^(1)^ and *Z*^(1)^ be the same as in Definition 1.Then, the gray differential equation

$$x^{\left(1\right)}\left(k\right)+az^{\left(1\right)}\left(k\right)=b$$

is referred to as the even form of model GM(1,1) (EGM(1,1) for short). If $\hat{a}={\left[a,b\right]}^{T}$ is a sequence of parameters, and

$$Y=\left[\begin{array}{c} x^{\left(1\right)}\left(2\right)\\ x^{\left(1\right)}\left(3\right)\\ \vdots \\ x^{\left(1\right)}\left(n\right) \end{array}\right],\;B=\left[\begin{array}{cc} -z^{\left(1\right)}\left(2\right) & 1\\ -z^{\left(1\right)}\left(3\right) & 1\\ \vdots & \vdots \\ -z^{\left(1\right)}\left(n\right) & 1 \end{array}\right]$$

then the least squares estimate sequence of the gray differential equation *x*^(1)^(*k*)+*az*^(1)^(*k*) = *b* satisfies the following:

$$\hat{a}={\left[a,b\right]}^{T}={\left(B^{T}B\right)}^{-1}B^{T}Y$$

**Definition 3.**[30]Let *X*^(0)^, *X*^(1)^ and $\hat{a}$ be the same as in Definition 1 and Definition 2.Then,thefollowingdifferential equation:

$$\frac{dx^{\left(1\right)}}{dt}+ax^{\left(1\right)}\left(k\right)=b$$

is called the whitenization (or image) equation of the gray differential equation *x*^(1)^(*k*)+*az*^(1)^(*k*) = *b*.

**Theorem 1**. [30]Let *X*^(0)^,*X*^(1)^ and $\hat{a}$ be the same as in Definition 1 and Definition 2.Then,the time response sequence of the EGM (1, 1) model is as follows:

$${\hat{x}}^{\left(1\right)}\left(k\right)=\left({\hat{x}}^{\left(0\right)}\left(1\right)-\frac{b}{a}\right)e^{-a\left(k-1\right)}+\frac{b}{a},k=2,3,\cdots ,n$$

In addition, the time response formula of ${\hat{x}}^{\left(0\right)}\left(k\right)$ can be given by the following:

$${\hat{x}}^{\left(0\right)}\left(k\right)={\hat{x}}^{\left(1\right)}\left(k\right)-{\hat{x}}^{\left(1\right)}\left(k-1\right)=\left(1-e^{a}\right)\left({\hat{x}}^{\left(0\right)}\left(1\right)-\frac{b}{a}\right)e^{-a\left(k-1\right)},k=2,3,\cdots ,n$$

**Table 2:** Symbols and their meanings[4].

Index	Symbol	Meaning
1	*X*^(0)^	An original time sequence
2	*X*^(1)^	1-Accumulation Generation Operator (AGO) sequence of *X*^(0)^
3	*Z*^(1)^	The mean sequence generated by consecutive neighbors of *X*^(1)^
4	*X*^(0)^*D*	1-Weighted Average Weakening Buffer Operator sequence of *X*^(0)^
6	${\hat{X}}^{\left(0\right)}$	The simulation time sequence of *X*^(0)^
6	*ε*	The error sequence of *X*^(0)^
7	Δ	The relative simulation percentage error (RSPE) of simulation sequence ${\hat{X}}^{\left(0\right)}$
8	*ρ*(*k*)	The smoothness ratio of sequence *X*

The accumulation generation is a vital step when building a gray prediction model. The size of the order of the accumulation generation is an important parameter that influences the simulation and prediction performances of gray prediction models. From the modeling process, the order of the accumulation generation for the EGM(1,1)model is fixed at ‘1’ (i.e., 1-AGO). Essentially, this is a simplification. Actually, the size of the order of the accumulation generation should satisfy the least mean relative simulation errors of the EGM(1,1)model. Then, the order is not always ‘1’, and may be a fraction or other integer. In recent years, study findings about the order of gray prediction models have appeared. In this paper, particle swarm optimization (PSO) is used to optimize the order of the EGM(1,1)model and resolve the imperfection of the order being fixed at ‘1’.

The parameters *a* and *b* of the EGM(1,1)model are estimated using the gray differential equation. However, *a* and *b* act as the parameters of the whitenization equation of the EGM(1,1)model (i.e., Equation(6)). The ‘misplaced replacement’ of the model parameters because of the conversion from the gray difference equation to the gray differential equation is the root cause of the poor performance of the EGM(1,1)model. In order to ensure the consistency of the model parameters, we directly estimate parameters *a* and *b* according to the gray differential equation of the EGM(1,1)model.

## An improved EGM(1,1) model, EGM(1,1,r)

In this section, the newly improved EGM(1,1) model with the optional order of the gray accumulation generation is established, and the time response formula of ${\hat{x}}^{\left(0\right)}\left(k\right)$ is directly deduced from the gray differential equation. After this, the above two operations resolve the two imperfections of the traditional EGM(1,1)model that was introduced in Section 2. The new EGM(1,1) model is abbreviated as EGM(1,1,*r*).

**Definition 4.**Let *X*^(0)^ = (*x*^(0)^(1),*x*^(0)^(2),⋯,*x*^(0)^(*n*)) be a nonnegative sequence, where *x*^(0)^(*k*)≥0, and *k* = 1,2,⋯,*n*. Then, *X*^(*r*)^ is the *r*-AGO sequence of *X*^(0)^ as follows:

$$X^{\left(r\right)}=\left(x^{\left(r\right)}\left(1\right),x^{\left(r\right)}\left(2\right),\cdots ,x^{\left(r\right)}\left(n\right)\right)$$

where

$$x^{\left(r\right)}\left(k\right)=\sum_{i=1}^{k}\frac{\Gamma \left(r+k-i\right)}{\Gamma \left(k-i+1\right)\Gamma \left(r\right)}x^{\left(0\right)}\left(i\right),\;k=1,2,\cdots ,n$$

In addition, *Z*^(*r*)^ is the mean generated sequence of consecutive neighbors of *X*^(*r*)^ as follows:

$$Z^{\left(r\right)}=\left(z^{\left(r\right)}\left(2\right),z^{\left(r\right)}\left(3\right),\cdots ,z^{\left(r\right)}\left(n\right)\right)$$

where

$$z^{\left(r\right)}\left(k\right)=0.5x^{\left(r\right)}\left(k\right)+0.5x^{\left(r\right)}\left(k-1\right),\;k=1,2,\cdots ,n$$

**Definition 5.**Let *X*^(0)^,*X*^(*r*)^ and *Z*^(*r*)^ be the same as in Definition 4.Then, the following gray differential equation

$$x^{\left(r-1\right)}\left(k\right)+az^{\left(r\right)}\left(k\right)=b$$

is referred to as the EGM(1,1)model with the order r of the accumulation generation (EGM(1,1,*r*) for short).

Similarly, the parameter vector $\hat{a}={\left[a,b\right]}^{T}$ of the EGM(1,1,*r*)model can be estimated using the least squares method from the gray differential equation *x*^(*r*−1)^(*k*)+*az*^(*r*)(^*k*) = *b* as follows:

$$\hat{a}={\left[a,b\right]}^{T}={\left(B^{T}B\right)}^{-1}B^{T}Y$$

where Y and B are as follow:

$$Y=\left[\begin{array}{c} x^{\left(r-1\right)}\left(2\right)\\ x^{\left(r-1\right)}\left(3\right)\\ \vdots \\ x^{\left(r-1\right)}\left(n\right) \end{array}\right],\;B=\left[\begin{array}{cc} -z^{\left(r\right)}\left(2\right) & 1\\ -z^{\left(r\right)}\left(3\right) & 1\\ \vdots & \vdots \\ -z^{\left(r\right)}\left(n\right) & 1 \end{array}\right]$$

From Definition 1,

$$x^{\left(r\right)}\left(k\right)=x^{\left(r\right)}\left(k-1\right)+x^{\left(r-1\right)}\left(k\right)\Rightarrow x^{\left(r-1\right)}\left(k\right)=x^{\left(r\right)}\left(k\right)-x^{\left(r\right)}\left(k-1\right)$$

Then,

$$Y=\left[\begin{array}{c} x^{\left(r-1\right)}\left(2\right)\\ x^{\left(r-1\right)}\left(3\right)\\ \vdots \\ x^{\left(r-1\right)}\left(n\right) \end{array}\right]=\left[\begin{array}{c} \left(r-1\right)x^{\left(0\right)}\left(1\right)+x^{\left(0\right)}\left(2\right)\\ \frac{r\left(r-1\right)}{2}x^{\left(0\right)}\left(1\right)+\left(r-1\right)x^{\left(0\right)}\left(2\right)+x^{\left(0\right)}\left(3\right)+\\ \vdots \\ \sum_{i=1}^{n}\frac{\Gamma \left(r+n-i\right)}{\Gamma \left(n-i+1\right)\Gamma \left(r\right)}x^{\left(0\right)}\left(i\right)-\sum_{i=1}^{n-1}\frac{\Gamma \left(r+n-1-i\right)}{\Gamma \left(n-i\right)\Gamma \left(r\right)}x^{\left(0\right)}\left(i\right) \end{array}\right]$$

$$B=\left[\begin{array}{cc} -z^{\left(r\right)}\left(2\right) & 1\\ -z^{\left(r\right)}\left(3\right) & 1\\ \vdots & \vdots \\ -z^{\left(r\right)}\left(n\right) & 1 \end{array}\right]=\left[\begin{array}{cc} -\frac{1}{2}\left[\left(r+1\right)x^{\left(0\right)}\left(1\right)+x^{\left(0\right)}\left(2\right)\right] & 1\\ -\frac{1}{2}\left[\frac{r\left(r+3\right)}{2}x^{\left(0\right)}\left(1\right)+(r+1)x^{\left(0\right)}\left(2\right)+x^{\left(0\right)}\left(3\right)\right] & 1\\ \vdots & \vdots \\ -\frac{1}{2}\left[\sum_{i=1}^{n}\frac{\Gamma \left(r+n-i\right)}{\Gamma \left(n-i+1\right)\Gamma \left(r\right)}x^{\left(0\right)}\left(i\right)+\sum_{i=1}^{n-1}\frac{\Gamma \left(r+n-1-i\right)}{\Gamma \left(n-i\right)\Gamma \left(r\right)}x^{\left(0\right)}\left(i\right)\right] & 1 \end{array}\right]$$

**Theorem 2**. Let *X*^(0)^,*X*^(*r*)^ and *Z*^(*r*)^ be the same as in Definition 4.Then, the time response sequence ${\hat{x}}^{\left(r\right)}\left(k\right)$ and the simulation sequence ${\hat{x}}^{\left(0\right)}\left(k\right)$ of the EGM (1, 1, *r*) model are as follows:

$${\hat{x}}^{\left(r\right)}\left(k\right)=\delta_{1}^{\left(k-1\right)}x^{\left(r\right)}\left(1\right)+\sum_{i=0}^{k-2}\delta_{1}^{i}\delta_{2}$$

and

$${\hat{x}}^{\left(0\right)}\left(k\right)=\sum_{i=0}^{k-1}{\left(-1\right)}^{i}\frac{\Gamma \left(r+1\right)}{\Gamma \left(i+1\right)\Gamma \left(r-i+1\right)}\left[\delta_{1}^{\left(k-2\right)}x^{\left(r\right)}\left(1\right)+\sum_{i=0}^{k-3}\delta_{1}^{i}\delta_{2}\right],k=2,3,\cdots ,n$$

where

$$\delta_{1}=\frac{1-0.5a}{1+0.5a},\delta_{2}=\frac{b}{1+0.5a}$$

**Proof**: From Definition 4,

$$x^{\left(r-1\right)}\left(k\right)+0.5a\left(x^{\left(r\right)}\left(k\right)+x^{\left(r\right)}\left(k-1\right)\right)=b$$

Since *x*^(*r*−1)^(*k*) = *x*^(*r*)^(*k*)−*x*^(*r*)^(*k*−1), and *x*^(*r*)^(*k*)−*x*^(*r*)^(*k*−1)+0.5*ax*^(*r*)^(*k*)+0.5*ax*^(*r*)^(*k*−1) = *b*,

$$x^{\left(r\right)}\left(k\right)=\frac{1-0.5a}{1+0.5a}x^{\left(r\right)}\left(k-1\right)+\frac{b}{1+0.5a}$$

Let *δ*_1_ = (1−0.5*a*)/(1+0.5*a*);*δ*_2_ = *b*/(1+0.5*a*)

That is,

$$x^{\left(r\right)}\left(k\right)=\delta_{1}x^{\left(r\right)}\left(k-1\right)+\delta_{2}$$

From Equation above, when *k* = 2,*x*^(*r*)^(2) = *δ*_1_*x*^(*r*)^(1)+*δ*_2_ when *k* = 3,

$$x^{\left(r\right)}\left(3\right)=\delta_{1}x^{\left(r\right)}\left(2\right)+\delta_{2}=\delta_{1}\left[\delta_{1}x^{\left(r\right)}\left(1\right)+\delta_{2}\right]+\delta_{2}=\delta_{1}^{2}x^{\left(r\right)}\left(1\right)+\delta_{1}\delta_{2}+\delta_{2}$$

⋮

And when *k* = *t*

$$x^{\left(r\right)}\left(t\right)=\delta_{1}^{\left(t-1\right)}x^{\left(r\right)}\left(1\right)+\delta_{1}^{\left(t-2\right)}\delta_{2}+\delta_{1}^{\left(t-3\right)}\delta_{2}+\cdots +\delta_{1}^{0}\delta_{2}$$

Rewriting Equation above, we obtain the time response sequence of the EGM (1,1,r) model as follows:

$$x^{\left(r\right)}\left(t\right)=\delta_{1}^{\left(t-1\right)}x^{\left(r\right)}\left(1\right)+\sum_{i=0}^{t-2}\delta_{1}^{i}\delta_{2}$$

According to the property of the fractional order accumulation generation operator, the simulation sequence of ${\hat{x}}^{\left(0\right)}\left(k\right)$ can be given by the following:

$${\hat{x}}^{\left(0\right)}\left(k\right)={\left({\hat{x}}^{\left(r\right)}\right)}^{\left(-r\right)}\left(k\right)=\sum_{i=0}^{k-1}{\left(-1\right)}^{i}\frac{\Gamma \left(r+1\right)}{\Gamma \left(i+1\right)\Gamma \left(r-i+1\right)}{\hat{x}}^{\left(r\right)}\left(k-i\right),k=2,3,\cdots ,n$$

That is,

$${\hat{x}}^{\left(0\right)}\left(k\right)=\sum_{i=0}^{k-1}{\left(-1\right)}^{i}\frac{\Gamma \left(r+1\right)}{\Gamma \left(i+1\right)\Gamma \left(r-i+1\right)}\left[\delta_{1}^{\left(k-2\right)}x^{\left(r\right)}\left(1\right)+\sum_{i=0}^{k-3}\delta_{1}^{i}\delta_{2}\right],k=2,3,\cdots ,n$$

This completes the proof.

In this paper, we use PSO to optimize the order of the EGM(1,1,*r*) model, and the optimization order of the EGM(1,1,*r*) model is sought under the condition of the least mean relative simulative errors, as follows:

$$\min f\left(r\right)=\frac{1}{n-1}\sum_{k=2}^{n}\frac{\left|{\hat{x}}^{\left(0\right)}\left(k\right)-x^{\left(0\right)}\left(k\right)\right|}{x^{\left(0\right)}\left(k\right)},r\in R^{+}$$

The detailed search process for the optimization order for the EGM(1,1,*r*) model can be found in[5].

## Modeling the conditions and error test method of EGM(1,1,r)

**Definition 6.** [30] Assume that *X*^(0)^ = (*x*^(0)^(1),*x*^(0)^(2),⋯,*x*^(0)^(*n*)), *x*^(0)^(*k*)≥0,and *k* = 1,2,⋯*n*. Then, the following is referred to as the smoothness ratio of sequence *X*:

$$\rho \left(k\right)=\frac{x^{\left(0\right)}\left(k\right)}{\sum_{i=1}^{k-1}x^{\left(0\right)}\left(i\right)},k=3,4,\cdots n$$

The concept of the smoothness ratio reflects the smoothness of a sequence from a special angle. In particular, it uses the ratio *ρ*(*k*) of the *k-*th data value *x*(*k*) to the sum $\sum_{i=1}^{k-1}x\left(i\right)$ of the previous values to check whether or not the changes in the data points of *X* are stable. The more stable that the changes of the data points in sequence *X* are, the smaller the ratio *ρ*(*k*) is.

**Definition 7**.Let *ρ*(*k*) be the same as in Definition 6.A sequence *X*^(0)^ = (*x*^(0)^(1),*x*^(0)^(2),⋯,*x*^(0)^(*n*)), where *x*^(0)^(*k*)≥0 and *k* = 1,2,⋯*n*, is referred to as a quasi-smooth sequence if it satisfies the following conditions:

- *λ*(*k*) = *ρ*(*k*+1)/*ρ*(*k*),*k* = 3,4,⋯*n*−1 *λ*(*k*) = *ρ*(*k*+1)/*ρ*(*k*),*k* = 3,4,⋯*n*−1;
- *λ*(*k*)<1;
- *ρ*(*k*)∈[0,*ε*],*k* = 3,4,⋯*n*;
- *ε*<0.8.

Quasi-smooth conditions are very important criteria for determining whether a sequence can be used to build a gray model.

After applying the accumulation operator a few times, the general nonnegative quasi-smooth sequence will show a pattern of exponential growth with decreased randomness. The smoother that the original sequence is, the more obvious an exponential growth pattern in the first order accumulation generated sequence will appear.

**Definition 8.** Let *X*^(0)^ = (*x*^(0)^(1),*x*^(0)^(2),⋯,*x*^(0)^(*n*)) be an original data sequence, where *x*^(0)^(*k*)≥0 and *k* = 1,2,⋯,*n*, and let ${\hat{X}}^{\left(0\right)}=\left({\hat{x}}^{\left(0\right)}\left(1\right),{\hat{x}}^{\left(0\right)}\left(2\right),\cdots ,{\hat{x}}^{\left(0\right)}\left(n\right)\right)$ be the simulation data sequence of *X*^(0)^ with the EGM(1,1,*r*)model. Then, the error sequence of *X*^(0)^ is as follows:

ε=(ε(1),ε(2),⋯,ε(n))

where

$$\varepsilon \left(u\right)=x^{\left(0\right)}\left(u\right)-{\hat{x}}^{\left(0\right)}\left(u\right),u=1,2,\cdots ,n$$

The relative simulation percentage error (RSPE) of the simulation sequence ${\hat{X}}^{\left(0\right)}$ is as follows:

Δ=(Δ(1),Δ(2),⋯,Δ(n))

where

$$\Delta \left(u\right)=\left|\frac{\varepsilon \left(u\right)}{x^{\left(0\right)}\left(u\right)}\times 100\%\right|,u=1,2,\cdots ,n$$

The mean relative simulation percentage error (MRSPE) of the simulation sequence is as follows:

$$\bar{\Delta }=\frac{1}{n}\sum_{u=1}^{n}\Delta \left(u\right)$$

For a giventhreshold value *α* (the threshold is set according to the specific situation of the system), when $\bar{\Delta }<\alpha$ holds true, the gray model is said to be error-satisfactory.

## Forecasting China’s beef consumption during 2019–2025

**Fig 1:**
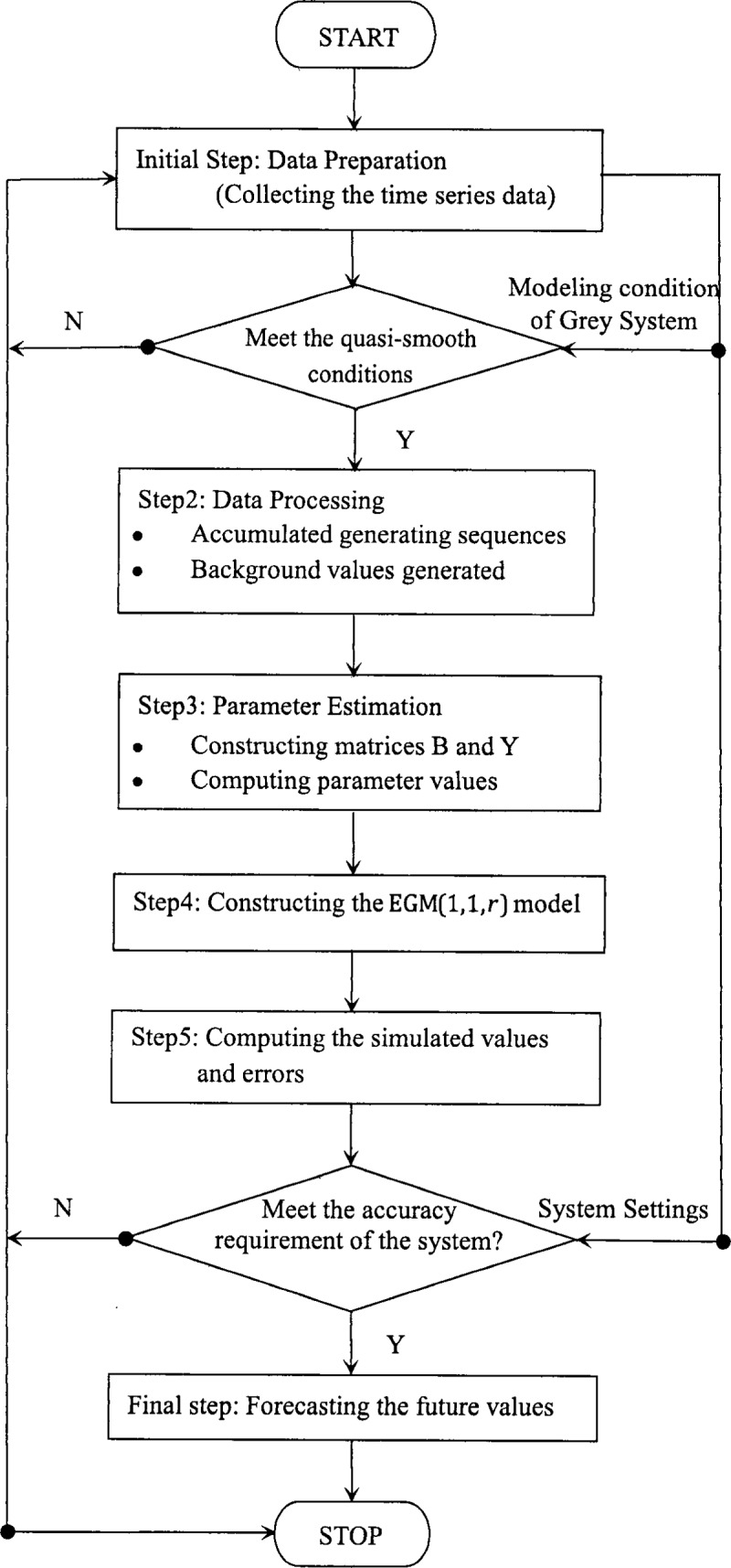
The flowchart of the EGM(1,1, r) model.

In this section, the EGM(1,1,*r*)model is employed to study the total beef consumption in China. The detailed modeling process of EGM(1,1,*r*) is introduced in Subsection 5.1.The simulation and prediction performances of EGM(1,1,*r*) are analyzed and compared with other models in Subsection 5.2. Finally, we use the EGM(1,1,*r*)model to forecast the total beef consumption in China from 2019 to 2025, and the predicted data are given in Subsection 5.3.To have a full understand of the research methods and improved model in this paper, we draw a flowchart as follows. (Fig 1)

## Modeling

### (1) Checking the quasi-smooth conditions before modeling

According to Definition 6, a sequence can be used to build the EGM(1,1,r) model only when it meets the quasi-smooth conditions. The total beef consumption in China from 1991 to 2015 is shown in Table 3.

**Table 3:** The total beef consumption in China from 1991 to 2015. (Unit: Ten thousand tons).

*k*	Year	Consumption	*k*	Year	Consumption	*k*	Year	Consumption	*k*	Year	Consumption
1	1991	131.3	8	1998	472.7	15	2005	561.4	22	2012	668.0
2	1992	172.9	9	1999	501.7	16	2006	569.2	23	2013	705.2
3	1993	218.4	10	2000	510.0	17	2007	606.5	24	2014	729.7
4	1994	303.6	11	2001	505.2	18	2008	608.0	25	2015	749.6
5	1995	405.1	12	2002	521.4	19	2009	634.0			
6	1996	345.7	13	2003	541.5	20	2010	652.0			
7	1997	432.3	14	2004	556.6	21	2011	644.9			

The ratios *ρ*(*k*) and *λ*(*k*) are computed according to Definitions 6–7, and the results are shown in Table 4, as follows.

**Table 4:** The values of *ρ*(*k*) and *λ*(*k*) from Equation (16) and Definition 7.

*k*	*ρ*(*k*)	*λ*(*k*)	*k*	*ρ*(*k*)	*λ*(*k*)	*k*	*ρ*(*k*)	*λ*(*k*)	*k*	*ρ*(*k*)	*λ*(*k*)
3	0.718	-	9	0.202	0.86	15	0.100	0.909	21	0.070	0.921
4	0.581	0.809	10	0.171	0.847	16	0.092	0.920	22	0.068	0.971
5	0.490	0.843	11	0.145	0.848	17	0.090	0.978	23	0.067	0.985
6	0.281	0.573	12	0.130	0.897	18	0.083	0.922	24	0.065	0.970
7	0.274	0.975	13	0.120	0.923	19	0.080	0.964	25	0.062	0.954
8	0.235	0.858	14	0.110	0.917	20	0.076	0.950			

It is obvious from Table 4 that *ρ*(*k*)<0.8(*k* = 3,4,…,25) and *λ*(*k*)<1(*k* = 4,5,…,25) are both true. Therefore, the data in Table 3 satisfy the modeling condition of the gray prediction model. Next, we use the EGM(1,1,*r*) model to simulate and forecast the total beef consumption in China.

### (2) Computing and optimizing parameters a, b and r

We use MATLAB to write the calculation program of the EGM(1,1,*r*) model, and apply Particle Swarm Optimization (PSO) to optimize the order ***r*** of EGM(1,1,*r*). The parameters’ values are as follows:

*a* = 0.011767, *b* = 127.481369;
*δ*_1_ = 0.988301, *δ*_2_ = 126.735694; and
*r* = 0.436213.

### (3) Building the EGM(1,1,*r*)model

According to Theorem 2 and the parameters *δ*_1_,*δ*_2_ and *r*, the response sequence ${\hat{x}}^{\left(r\right)}\left(k\right)$ and the simulation sequence ${\hat{x}}^{\left(0\right)}\left(k\right)$ of the EGM (1, 1, *r*) model are as follows:

$${\hat{x}}^{\left(r\right)}\left(k\right)=131.3\times 0.988301^{\left(k-1\right)}+\sum_{i=0}^{k-2}126.735694\times 0.988301^{i}$$

and

$${\hat{x}}^{\left(0\right)}\left(k\right)=\sum_{i=0}^{k-1}{\left(-1\right)}^{i}\frac{\Gamma \left(1.436213\right)}{\Gamma \left(i+1\right)\Gamma \left(1.436213-i\right)}\cdot {\hat{x}}^{\left(r\right)}\left(k\right),k=2,3,\cdots ,n$$

### Comparing and analyzing the performances of the models

In this subsection, we detail the use of the EGM(1,1,*r*) model to simulate the total beef consumption in China during the period from 1991–2015. To verify the performance of EGM(1,1,*r*), we compare the MRSPE of EGM(1,1,*r*) to that of the most commonly implemented gray models, including the GM(1,1), GM(1,1) and SAIGM models.

The simulated values ${\hat{x}}^{\left(0\right)}\left(k\right)$, the residual values *ε*(*k*), the RSPE Δ_*k*_, and the MRSPE of the four models are presented in Table 5.

**Table 5:** Simulation values and MRSPEs of the four models for China’s beef consumption.

Actual Value *x*^(0)^(*k*)	EGM(1,1,r)		GM(1,1)		DGM(1,1)		SAIGM	
	Simulation value ${\hat{x}}^{\left(0\right)}\left(k\right)$	Simulation error Δ_*k*_(%)	Simulation value ${\hat{x}}^{\left(0\right)}\left(k\right)$	Simulation error Δ_*k*_(%)	Simulation value ${\hat{x}}^{\left(0\right)}\left(k\right)$	Simulation error Δ_*k*_(%)	Simulation value ${\hat{x}}^{\left(0\right)}\left(k\right)$	Simulation error Δ_*k*_(%)
131.3	-	-	-	-	-	-	-	-
172.9	199.22	15.22	335.28	93.92	335.9	94.27	210.28	21.62
218.4	252.20	15.48	347.89	59.29	348.5	59.57	255.61	17.04
303.6	296.70	2.27	360.98	18.90	361.57	19.09	297.4	2.04
405.1	335.58	17.16	374.56	7.54	375.13	7.40	335.94	17.07
345.7	370.35	7.13	388.65	12.42	389.2	12.58	371.47	7.45
432.3	401.92	7.03	403.27	6.72	403.79	6.59	404.23	6.49
472.7	430.90	8.84	418.43	11.48	418.94	11.37	434.44	8.09
501.7	457.71	8.77	434.17	13.46	434.65	13.36	462.29	7.86
510.0	482.67	5.36	450.50	11.67	450.95	11.58	487.97	4.32
505.2	506.02	0.16	467.45	7.47	467.86	7.39	511.65	1.28
521.4	527.96	1.26	485.03	6.97	485.41	6.90	533.49	2.32
541.5	548.63	1.32	503.28	7.06	503.62	7.00	553.62	2.24
556.6	568.16	2.08	522.21	6.18	522.5	6.13	572.18	2.8
561.4	586.65	4.50	541.85	3.48	542.1	3.44	589.3	4.97
569.2	604.20	6.15	562.23	1.22	562.43	1.19	605.08	6.3
606.5	620.88	2.37	583.38	3.81	583.52	3.79	619.63	2.16
608.0	636.75	4.73	605.32	0.44	605.41	0.43	633.05	4.12
634.0	651.89	2.82	628.09	0.93	628.11	0.93	645.42	1.8
652.0	666.32	2.20	651.72	0.04	651.67	0.05	656.82	0.74
644.9	680.11	5.46	676.23	4.86	676.11	4.84	667.34	3.48
668.0	693.29	3.79	701.67	5.04	701.47	5.01	677.04	1.35
705.2	705.9	0.10	728.06	3.24	727.78	3.20	685.98	2.73
729.7	717.96	1.61	755.44	3.53	755.07	3.48	694.23	4.86
749.6	729.52	2.68	783.86	4.57	783.39	4.51	701.83	6.37
MRSPE(%)		5.35		12.26		12.25		5.82

It can be seen from Table 5 that the MRSPE of the EGM(1,1,*r*) model is only 5.35%, which is the lowest among the four models, and the second lowest MRSPE is that of the SAIGM model, at 5.82%. The performance of GM(1,1) is similar to that of DGM(1,1), and the MRSPE(12.26%) of GM(1,1) is more than two times that of EGM(1,1,*r*). Hence, this shows that the performance of EGM(1,1,*r*) is the best among the four models.

To better show the performance differences among them, we draw four scatter line figures regarding the actual data and simulated data from Table 5 for the above four models, as follows.

**Fig 2:**
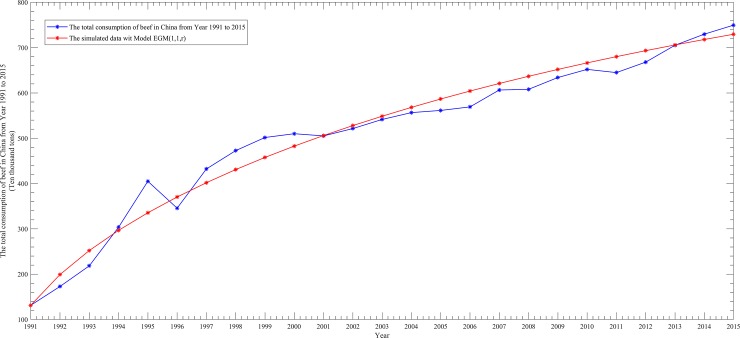
Curves of the actual data and simulated data of the EGM(1,1,*r*)model.

**Fig 3:**
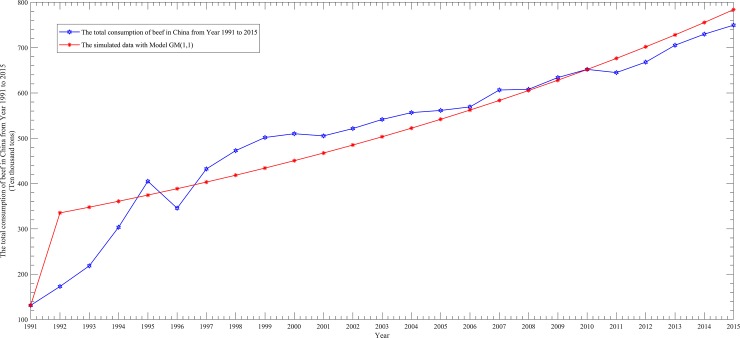
Curves of the actual data and simulated data of the GM(1,1)model.

**Fig 4:**
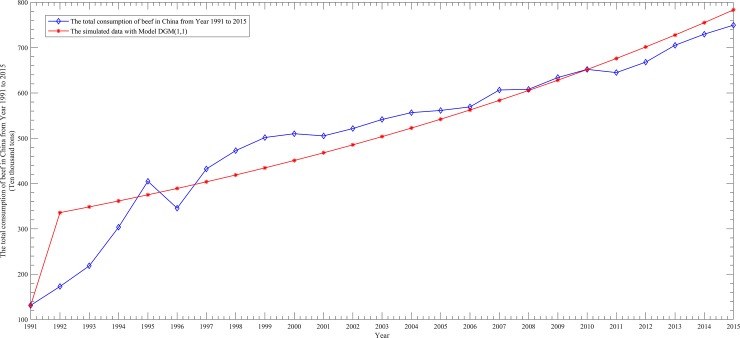
Curves of the actual data and simulated data of the DGM(1,1)model.

**Fig 5:**
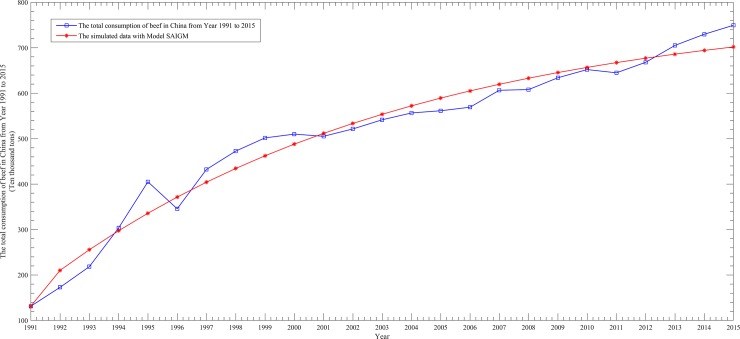
Curves of the actual data and simulated data of the SAIGMmodel.

From Figs 2–5, it can be seen that the simulation curve of the EGM(1,1,*r*) model is the closest to that of the actual data among the four models, which again illustrates the fact that EGM(1,1,*r*) has the best simulation performance. However, a prediction model cannot be judged as being good or bad based only on the MRSPE, since a good simulation performance does not necessarily mean that its predictive performance is also good. Hence, it is necessary to verify the predictive precision of a model before applying it to forecasting future data.

In this section, we use the above four models to forecast China’s total beef consumption in 2016, 2017and 2018 and compare their predictive errors. (China’s total beef consumptions during 2016, 2017and 2018are 767,794and 814, respectively. These numbers are from China’s statistics yearbook). The prediction data and errors for the four models are as follows:

- The prediction data of the EGM(1,1,*r*) model:
  ${\hat{x}}^{\left(0\right)}{\left(k\right)}_{2016}=740.59$, ${\hat{x}}^{\left(0\right)}{\left(k\right)}_{2017}=751.21$ and ${\hat{x}}^{\left(0\right)}{\left(k\right)}_{2018}=783.11$
  $\Delta_{2016}=\left|\frac{740.59-767}{767}\times 100\%\right|=3.44\%$
  $\Delta_{2017}=\left|\frac{751.21-794}{794}\times 100\%\right|=5.39\%$ and
  $\Delta_{2018}=\left|\frac{783.11-814}{814}\times 100\%\right|=3.79\%$
  $\Delta_{\mathrm{EGM}(1,1,r)}=\frac{\Delta_{2016}+\Delta_{2017}+\Delta_{2018}}{3}=4.21\%$
- The prediction data of the GM(1,1) model:
  ${\hat{x}}^{\left(0\right)}{\left(k\right)}_{2016}=813.34$, ${\hat{x}}^{\left(0\right)}{\left(k\right)}_{2017}=843.94$ and ${\hat{x}}^{\left(0\right)}{\left(k\right)}_{2018}=875.68$
  $\Delta_{2016}=\left|\frac{813.34-767}{767}\times 100\%\right|=6.04\%$
  $\Delta_{2017}=\left|\frac{843.94-794}{794}\times 100\%\right|=6.29\%$ and
  $\Delta_{2018}=\left|\frac{875.68-814}{814}\times 100\%\right|=7.58\%$
  $\Delta_{\mathrm{GM}(1,1)}=\frac{\Delta_{2016}+\Delta_{2017}+\Delta_{2018}}{3}=6.64\%$
- The prediction data of the DGM(1,1) model:
  ${\hat{x}}^{\left(0\right)}{\left(k\right)}_{2016}=812.77$, ${\hat{x}}^{\left(0\right)}{\left(k\right)}_{2017}=843.25$ and ${\hat{x}}^{\left(0\right)}{\left(k\right)}_{2018}=874.88$
  $\Delta_{2016}=\left|\frac{812.77-767}{767}\times 100\%\right|=5.97\%$
  $\Delta_{2017}=\left|\frac{843.25-794}{794}\times 100\%\right|=6.20\%$ and
  $\Delta_{2018}=\left|\frac{874.88-814}{814}\times 100\%\right|=7.48\%$
  $\Delta_{\mathrm{DGM}(1,1)}=\frac{\Delta_{2016}+\Delta_{2017}+\Delta_{2018}}{3}=6.55\%$
- The prediction data of the SAIGM model:
  ${\hat{x}}^{\left(0\right)}{\left(k\right)}_{2016}=708.84$, ${\hat{x}}^{\left(0\right)}{\left(k\right)}_{2017}=715.30$ and ${\hat{x}}^{\left(0\right)}{\left(k\right)}_{2018}=721.26$
  $\Delta_{2016}=\left|\frac{708.84-767}{767}\times 100\%\right|=7.58\%$
  $\Delta_{2017}=\left|\frac{715.30-794}{794}\times 100\%\right|=9.91\%$ and
  $\Delta_{2018}=\left|\frac{721.26-814}{814}\times 100\%\right|=11.39\%$
  $\Delta_{\mathrm{SAIGM}}=\frac{\Delta_{2016}+\Delta_{2017}+\Delta_{2018}}{3}=9.63\%$

From the above calculation results, we see that the prediction error of the EGM(1,1,*r*) model is the smallest among the four models. The second smallest is that of DGM(1,1), which is slightly better than that of GM(1,1); and the SAIGM model is the worst. From Table 5, the simulation performance of SAIGM is second only to EGM(1,1,*r*) and far superior to those of DGM(1,1) and GM(1,1). However, the prediction performance of SAIGM is the worst among the four models. It shows that having a good simulation performance cannot guarantee that a model has a good prediction performance.

### Forecasting the total consumption of beef in China

Synthesizing the above conclusions, we see that both the simulation and prediction errors of EGM(1,1,*r*) are the smallest among the four models. Therefore we apply this new model to forecasting China’s total beef consumption. The results are shown in Table 6, as follows.

**Table 6:** Predicted data for China’s total beef consumption from 2019 to 2025 (Unit: Ten thousand tons).

*k*	Year	Consumption	*k*	Year	Consumption	*k*	Year	Consumption	*k*	Year	Consumption
29	2019	794.71	31	2021	816.80	33	2023	837.55	35	2025	857.04
30	2020	805.93	32	2022	827.34	34	2024	847.44			

From Table 6, we see that the total beef consumption in China is predicted to increase to 857.04 ten thousand tons in 2025, which is more than six times the consumption in 1991; and the annual average growth rate will be approximately 15.82%. However, for the past ten years, the growth rate has been approximately 4% and has been smooth on the whole, although the total beef consumption in China is very large. China will import more than 1.0 million tons of beef next year, based on actual domestic beef production and predicted beef consumption of proposed model. Beef imports account for about 15 percent of total beef consumption in China. Therefore, according to the above prediction results and analysis, the Chinese government can formulate relevant policies and measures in order to ensure the balance between the supply and demand with respect to Chinese beef consumption.

## Conclusions

Scientifically and effectively forecasting the total beef consumption in China is of great significance for promoting the effective supply of Chinese beef. However, there are many factors that affect beef consumption in China, and they show the typical characteristics of ‘Gray Factor White Result’. Hence, it is difficult for the traditional mathematical statistical model to effectively simulate and predict Chinese beef consumption and compared with GM(1,1),DGM(1,1),SAIGM models. The MRSPE of the EGM(1,1,*r*) model is only 5.35%, which is the lowest among the four models, and the second lowest MRSPE is that of the SAIGM model, at 5.82%. The performance of GM(1,1) is similar to that of DGM(1,1), and the MRSPE(12.26%) of GM(1,1) is more than two times that of EGM(1,1,*r*). Moreover, the prediction error of the EGM(1,1,*r*) model is only 4.21%, which is the smallest among the four models. The second smallest is that of DGM(1,1), which is slightly better than that of GM(1,1); and the SAIGM model is the worst. So a good simulation performance does not necessarily mean that its predictive performance is also good. It is necessary to verify the predictive precision of a model before applying it to forecasting future data. To this end, an improved gray system model was used to simulate and predict Chinese beef consumption. The results showed that the new model is superior to other gray forecasting models in both simulation and prediction performance. Finally, the EGM(1,1,*r*) model was used to predict the total beef consumption in China for the period of 2019–2025. The results showed that the total beef consumption in China will keep growing for a long time. By 2025, the total beef consumption in China is predicted to reach 857.04 ten thousand tons, which is more than 6 times the total Chinese beef consumption in 1991. Exploring the influencing factors and trend prediction of beef prices in China is the next research objective of the project team.
